# Pneumatosis Cystoides Intestinalis Identified on Screening Colonoscopy With Associated Pneumoperitoneum

**DOI:** 10.7759/cureus.9512

**Published:** 2020-08-01

**Authors:** Matthew J Lommen, Omar Zineldine, Tej I Mehta, Logan E Radtke, Oluwagbenga Serrano

**Affiliations:** 1 Radiology, University of South Dakota Sanford School of Medicine, Sioux Falls, USA; 2 Surgery, University of South Dakota Sanford School of Medicine, Sioux Falls, USA; 3 Gastroenterology, Good Samaritan Hospital, Vincennes, USA

**Keywords:** pneumatosis cystoides intestinalis, colonoscopy, pneumoperitoneum, pneumatosis intestinalis

## Abstract

Pneumatosis cystoides intestinalis (PCI) is defined by the presence of gas within the bowel wall. It is often asymptomatic and usually benign but may be associated with significant morbidity and mortality. In this patient, PCI was found incidentally on screening colonoscopy, and biopsy of the affected mucosa resulted in deflation of a cyst. Pneumoperitoneum was then identified on subsequent CT. Because pneumoperitoneum is associated with bowel perforation in most cases, it is often treated as an indication for operation. This case of benign and asymptomatic pneumoperitoneum was managed conservatively without complications. Clinicians should be able to identify PCI as a potentially benign finding on colonoscopy as well as a potentially benign cause of pneumoperitoneum. This understanding presents an opportunity to avoid the unnecessary morbidity and costs associated with surgical exploration or additional endoscopic procedures.

## Introduction

Pneumatosis cystoides intestinalis (PCI) is a rare finding of intramural gas within the walls of the small and/or large intestine. PCI is not itself a diagnosis but is a radiographic or physical finding that may be associated with an underlying disease process [1]. An autopsy series reported a prevalence of 0.03% in the general population, though the true prevalence of PCI remains unknown [2]. It may be associated with severe disease such as necrotizing enterocolitis or identified incidentally on radiographic, endoscopic, or pathologic examination [2,3]. Rarely, PCI represents a cause of benign pneumoperitoneum or air within the peritoneal cavity [4]. Despite current knowledge and hypotheses regarding the pathogenesis, epidemiology, presentation, diagnosis, and management of PCI, there remains a lack of multicenter, prospective clinical trials assessing factors contributing to diagnosis and management of benign versus pathologic PCI.

We present a case of PCI identified incidentally on screening colonoscopy with concurrent pneumoperitoneum present on CT. Our objective is a contribution to, and review of, current literature regarding this topic.

## Case presentation

A 65-year-old woman presented for her first screening colonoscopy. Her medical history was significant for morbid obesity, anxiety, hypertension, and a 46 pack-year history of tobacco use. She had previously undergone a laparoscopic cholecystectomy, ventral hernia repair, and cylindroma excision but had no surgeries in the 12 months prior to her presentation. Her medications at the time included hydrochlorothiazide and losartan. She had no family history of colon cancer or any recent significant gastrointestinal symptoms.

**Figure 1:**
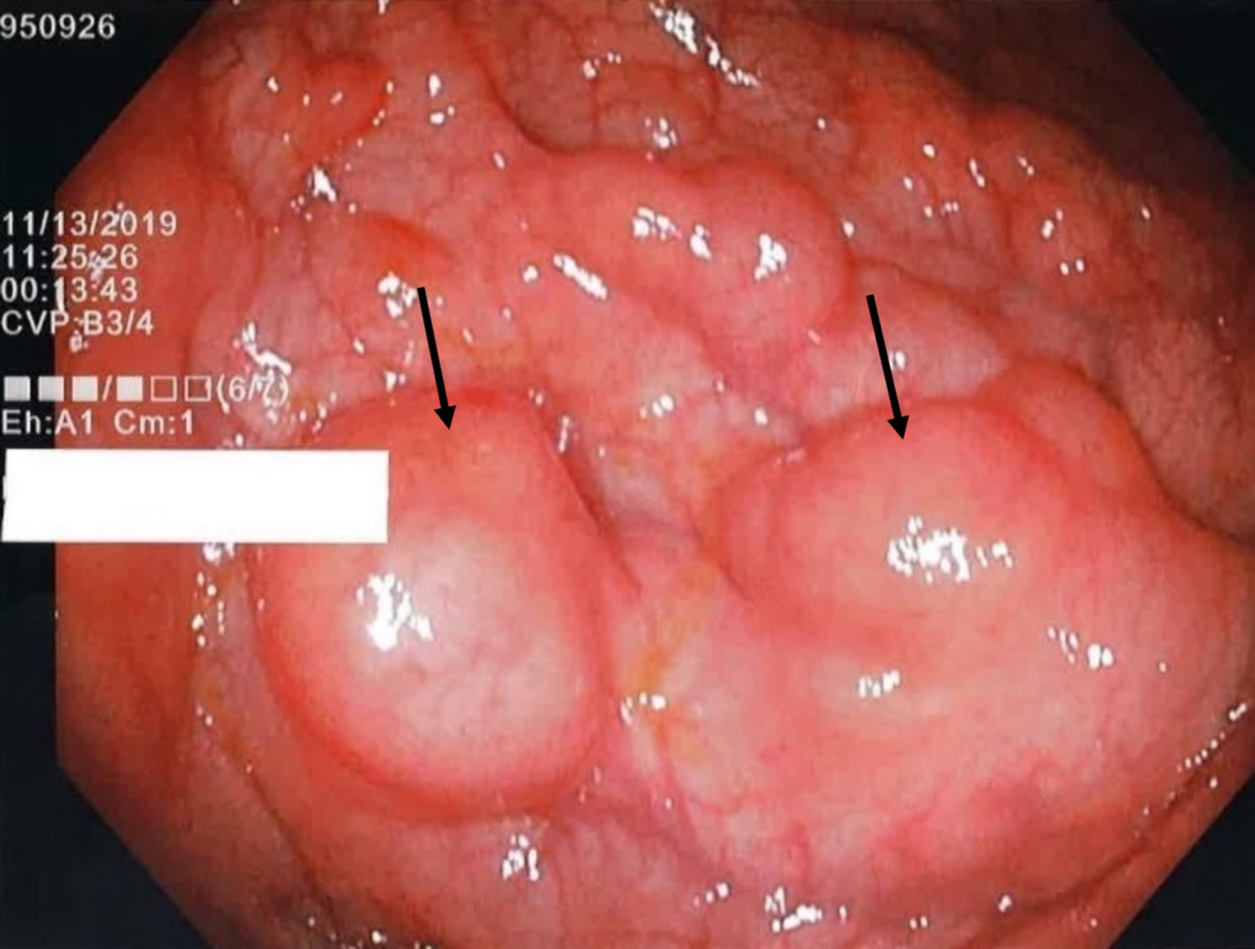
Colonoscopy findings of multiple masses at the splenic flexure consistent with PCI PCI: pneumatosis cystoides intestinalis; black arrows: cystic lesions arising from colonic mucosa

The patient’s colonoscopy revealed polypoid grape-like masses protruding through the mucosa, consistent with PCI at the splenic flexure as well as small, benign colonic polyps at other areas of the colon (Figure 1).

**Figure 2:**
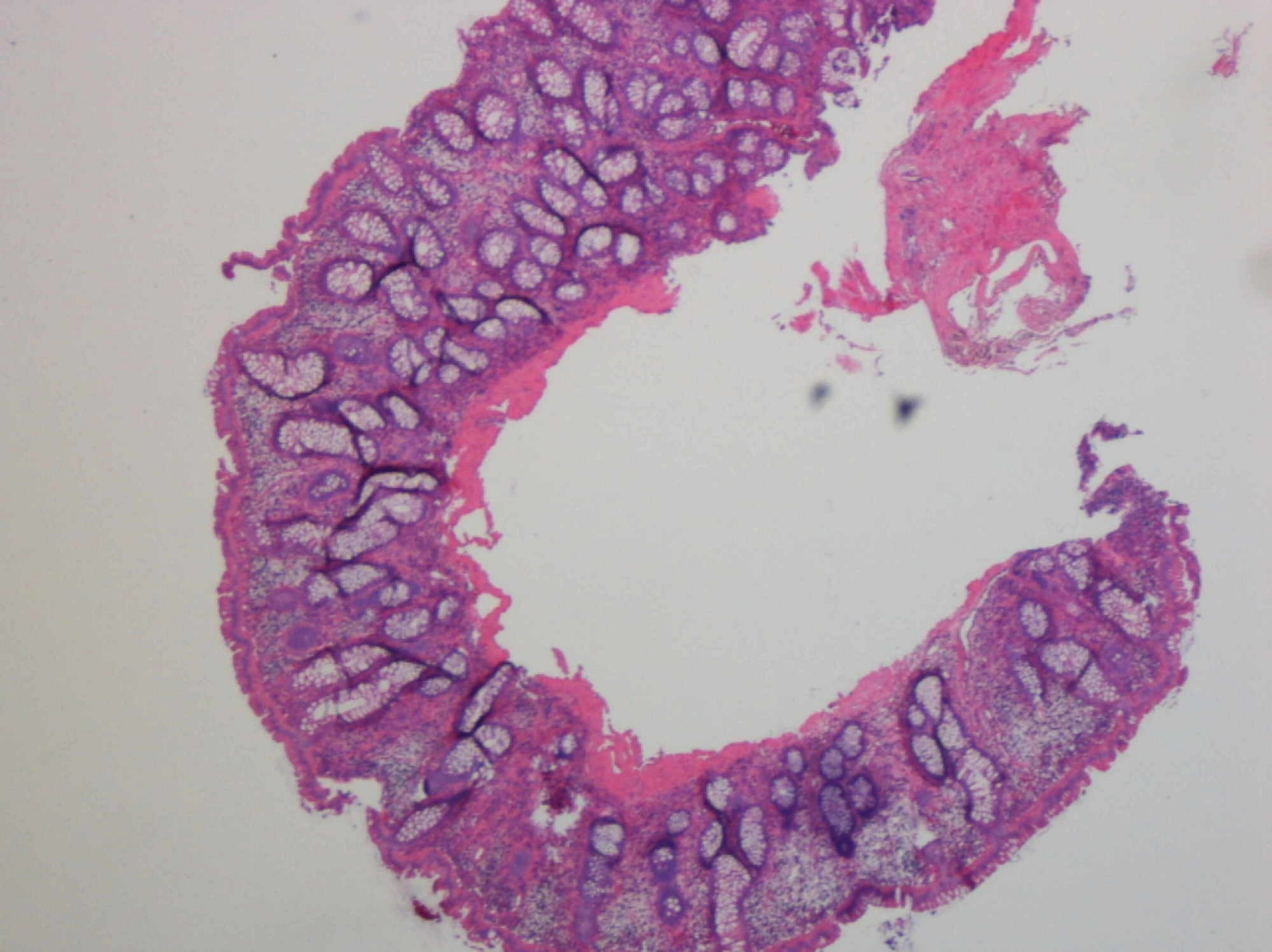
Histologic findings of submucosal cysts lined by multinucleated giant cells on sigmoid biopsy

Biopsy of one of the grape-like masses at the splenic flexure was performed resulting in its visible rupture and collapse. Histologic examination of the biopsy demonstrated empty, submucosal cysts lined by multinucleated giant cells (Figure 2). 

**Figure 3:**
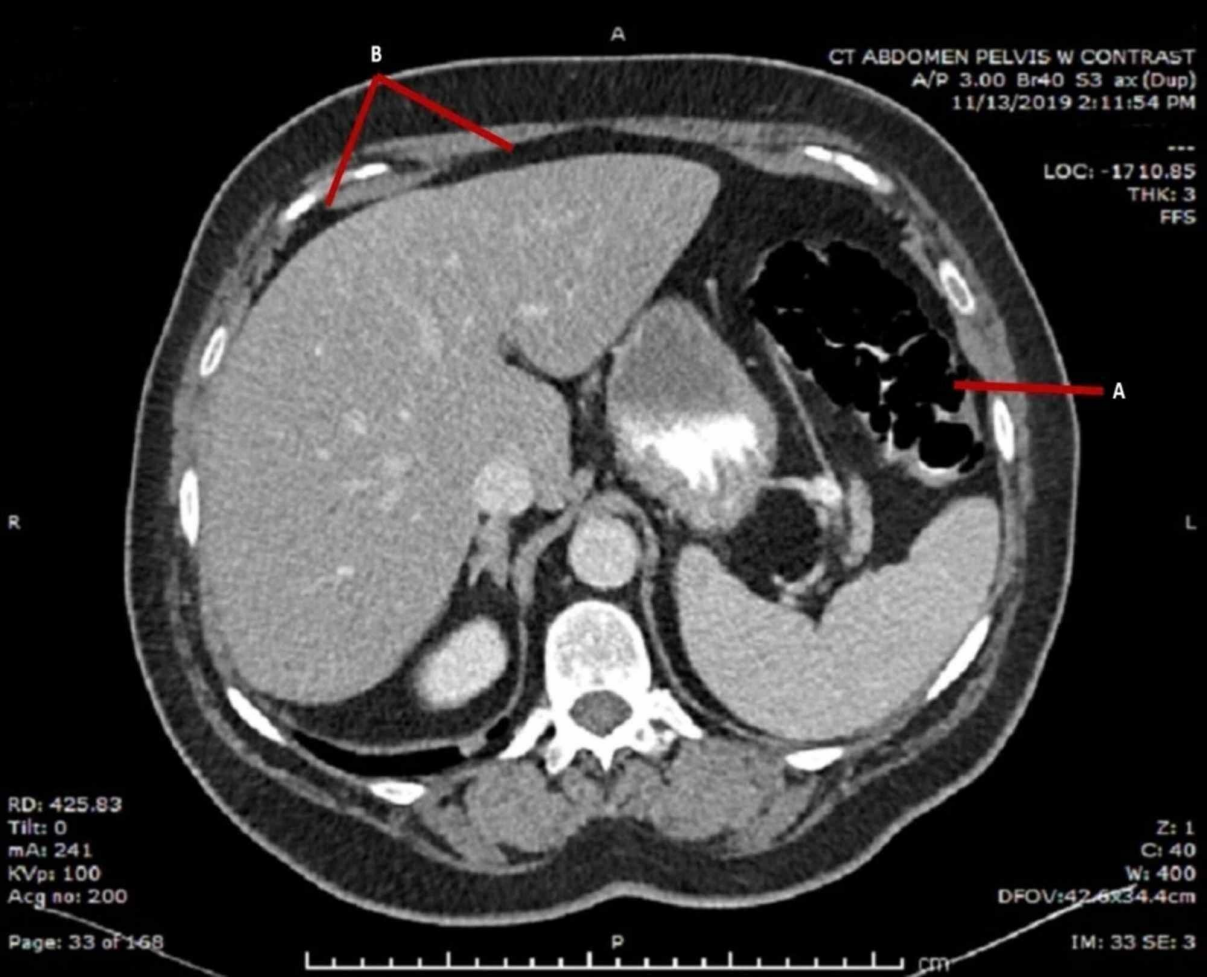
Post-colonoscopy CT abdomen revealing findings associated with pneumatosis cystoides intestinalis (PCI) and pneumoperitoneum (A) Air-filled cystic spaces within the gastrointestinal wall suggesting PCI; (B) findings of intra-abdominal free air.

Post-procedure CT images showed multiple, thin-walled, air-filled submucosal cysts with evidence of intraperitoneal free air (Figure 3). Given the patient was vitally stable without guarding or rigidity on exam, the decision was made to manage the patient conservatively with monitoring and serial exams. She remained asymptomatic and did not require surgical intervention. She was discharged home and has remained asymptomatic after three months of follow-up. 

## Discussion

The pathogenesis of PCI remains incompletely understood, despite several hypotheses. These hypotheses suggest mucosal damage in combination with increased intraluminal pressure secondary to mechanical causes, bacterial production, or pulmonary gas traveling along the perivascular spaces of mesenteric vessels [1,5,6]. Approximately 15% of PCI cases have been categorized as idiopathic, or primary, in origin, while up to 85% of cases are secondary [7]. Disorders associated with PCI include, but are not limited to, chronic pulmonary disease, acquired immunodeficiency, history of transplant, systemic lupus erythematosus, inflammatory bowel disease, intestinal ischemia, and trauma [8]. Many patients with PCI are asymptomatic but can present with abdominal discomfort, diarrhea, abdominal distension, nausea, vomiting, hematochezia, mucousy stool, and constipation [9]. Our patient was asymptomatic and had not been diagnosed with any known associated diseases, though she does have a 46 pack-year history of tobacco abuse. It may be reasonable to hypothesize a subclinical respiratory disorder that may have contributed to her development of PCI, yet the true cause remains unknown.

Identification of PCI may be increasing along with the use of CT in diagnosing abdominal pathology [5]. CT is the ideal imaging modality for diagnosis of PCI, and a classic finding includes intramural gas parallel to the bowel wall (Figure 3) [10,11]. Prior reviews had found PCI to more commonly affect the small intestine, but more recent studies have found a higher incidence in the colon (46%-61.8%), as was seen with our patient [12]. The small intestine is affected in 15.4%-27% of cases, and only in 2.9%-7.0% cases is PCI present in both the colon and small intestine [9,13,14].

Difficulty identifying patients with PCI secondary to a potentially deadly pathologic cause remains a barrier to management. A 2013 review found PCI to represent a benign finding in 60% of cases and was associated with significant morbidity and mortality in approximately 40% of cases. Their analysis found a lactate elevation of 2.0 or greater to be the strongest independent predictor for pathologic disease. This lactate elevation in combination with hypotension or vasopressor need was found to have a predictive probability of 93.2% for pathologic disease [5]. Lactate ≥2.0 at time of diagnosis has further been equated with a greater than 80% mortality rate, indicating a need for immediate surgical consultation and management [15].

Pneumoperitoneum, or free air in the abdominal cavity, is a concerning imaging finding that prompts immediate attention. It is estimated that 85%-95% of cases of pneumoperitoneum are associated with perforation of intra-abdominal viscus, and thus the tendency towards surgical management. In contrast, pneumoperitoneum found in association with PCI is often a benign finding but does not rule out viscus perforation [4]. This patient did undergo a colonoscopy, and while iatrogenic colonic perforation during colonoscopy is a possible explanation for the presence of pneumoperitoneum, it is quite rare with an estimated incidence of 0.12% [16]. Benign pneumoperitoneum following colonoscopy is thought to be rare or nonexistent with an estimated incidence of 0%-3% [17].

## Conclusions

As the number of screening colonoscopies continues to rise, recognition of PCI by experienced endoscopists will become increasingly important. Identification can avoid misdiagnosis of polyposis or neoplasm and reduce unnecessary additional diagnostic studies while also ensuring proper management and follow-up to rule out pathologic causes. Although it is uncommon, clinicians and radiologists should also be on the lookout for PCI as a cause of benign pneumoperitoneum. Asymptomatic patients without concerning findings on exam may be managed conservatively with serial exams and avoid the complications and costs associated with surgical exploration or additional endoscopic procedures.
